# Cascade‐To‐Single Layer Transformation of Multifunctional Multispectral Metasurfaces

**DOI:** 10.1002/nap2.70132

**Published:** 2026-05-11

**Authors:** Ihar Faniayeu, Ivan Fanyaev

**Affiliations:** ^1^ Department of Physics University of Gothenburg Gothenburg Sweden; ^2^ Department of Radiophysics and Electronics Francisk Skorina Gomel State University Gomel Belarus

## Abstract

Multifunctional metasurfaces are commonly realized using cascaded or few‐layer architectures, in which each layer performs a dedicated electromagnetic function. Although such designs offer increased degrees of freedom and high efficiency, they inevitably increase overall thickness and fabrication complexity. Achieving comparable multifunctional performance within a single‐layer metasurface therefore constitutes a more compact and practically attractive solution. Here, we show that a five‐function cascaded metasurface can be systematically collapsed into a single‐layer metasurface while preserving electromagnetic functionality and efficiency. The proposed design employs a helix‐inspired supercell architecture that integrates multiple frequency‐selective meta‐atoms within a single planar layer. Each meta‐atom operates independently at a designated frequency, enabling polarization‐selective reflection and transmission, circular‐polarization conversion, and absorption in an ultrathin layer. Full‐wave simulations confirm that the single‐layer metasurface reproduces the performance of its cascaded counterpart with high efficiency, polarization robustness, and angular stability across all operating bands. These results establish an approach for translating cascaded metasurface architectures into single‐layer implementations, providing a scalable pathway toward ultracompact multifunctional electromagnetic devices.

## Graphical abstract

A cascade‐to‐single‐layer transformation strategy is introduced to realize a passive multifunctional multispectral metasurface. Various electromagnetic functionalities, including polarization conversion in reflection and transmission, and absorption, are integrated within a single unit cell at different frequencies. The approach preserves performance while reducing structural complexity, offering a compact and robust alternative to multilayer and tunable designs for advanced RF applications.

## 1 Introduction

Metasurfaces, comprising planar arrays of subwavelength scattering elements, have emerged as a powerful platform for manipulating electromagnetic waves through ultrathin and lightweight structures [1, 2, 3]. In the radio‐frequency (RF) and microwave regimes, metasurfaces are particularly attractive, as conventional wave‐control components such as lenses [4, 5], polarizers [6, 7], absorbers [8, 9] and beamformers [10, 11] still dominate many systems, metasurfaces offer an especially attractive route to compact wave control because metallic patterning and multilayer laminations are comparatively low‐loss and scalable at centimeter‐to‐millimeter wavelengths. As a result, RF metasurfaces have rapidly evolved from demonstrating single functions (e.g., steering or polarization conversion at a single band) toward multifunctional devices that can manipulate phase, amplitude, polarization, and scattering response within the same aperture and often across multiple frequency bands [12, 13, 14, 15].

Driven by the growing demand for compact and integrated solutions in wireless communications, radar, and sensing systems, metasurface research has increasingly focused on multifunctional devices capable of performing several electromagnetic functions within a single platform [15, 16, 17]. A widely adopted approach to realize such multifunctionality relies on *cascaded* or few‐layer metasurfaces [18, 19, 20], where each layer is engineered to provide a distinct function, such as polarization rotation, circular polarization conversion, transmission or reflection control, or absorption operating often at different frequencies. By stacking multiple functional layers, cascaded metasurfaces significantly increase the available degrees of freedom and enable complex electromagnetic responses that are difficult to achieve with a single layer alone. It is worth briefly noting that the push toward multifunctional metasurfaces has also been vigorous in optics, where researchers have pursued multiplexing (by wavelength, polarization, and spatial mode) and multi‐task meta‐optics such as achromatic imaging and multi‐wavelength operation [21, 22, 23]. Studies at optical frequencies highlight a general principle that is equally applicable at RF: metasurface multifunctionality is governed by the available degrees of freedom, namely the number of independent geometric, material, structural, and control parameters that can be exploited to tailor multiple electromagnetic proper while maintaining high efficiency and practical manufacturability.

Few‐layer metasurfaces benefit from strong interlayer effects, including multiple‐wave interference and near‐field coupling, which enhance light–matter interactions and improve operational efficiency. Moreover, the combination of distinct functional layers allows the realization of advanced multifunctional responses within a single composite structure [24, 25, 26]. However, these advantages come at the cost of increased thickness, fabrication complexity, alignment sensitivity, and reduced compatibility with ultracompact or planar‐integrated architectures. From both a fundamental and practical perspective, achieving the same level of multifunctionality and efficiency within a *single‐layer* metasurface represents a highly desirable and more optimal solution.

Realizing such a transition from cascaded to single‐layer metasurfaces is nontrivial. In a single layer, the available degrees of freedom are inherently limited, and different functionalities must coexist within the same plane without introducing strong mutual coupling or performance degradation. In this context, single‐layer metasurfaces have primarily been investigated either for a restricted set of functionalities or in regimes where trade‐offs between efficiency and multifunctionality are explored in comparison with multilayer approaches [27, 28, 29, 30, 31, 32, 33]. At the same time, recent progress in reconfigurable and tunable metasurfaces [34, 35, 36] has enabled dynamic control of electromagnetic responses, albeit often at the expense of increased structural complexity, biasing networks, and additional losses. In contrast, static single‐layer designs offer a passive, compact, structurally simple, and cost‐effective alternative by integrating multiple functionalities within a single unit cell without external control.

Recent advances in reconfigurable and tunable metasurfaces have demonstrated dynamic control of electromagnetic responses, but often at the cost of increased structural complexity, biasing networks, and additional losses. In contrast, the proposed static single‐layer design provides a passive, compact, structurally simple, and cost‐effective solution that integrates multiple functionalities within a single unit cell, without requiring external control. This makes it particularly attractive for applications demanding robustness, simplicity, and reliable operation under high‐power conditions.

In this work, we demonstrate that a multifunctional cascaded metasurface can be systematically transformed into a *single‐layer* metasurface while preserving its electromagnetic functionality and high efficiency. Our approach is based on a helix‐based supercell architecture that spatially integrates multiple resonant meta‐atoms within a single planar layer as illustrated in Figure 1. Each meta‐atom is designed to operate independently at a designated frequency, enabling five distinct electromagnetic functions, including polarization‐selective reflection and transmission, circular polarization conversion, and absorption within one ultrathin metasurface.

**FIGURE 1:**
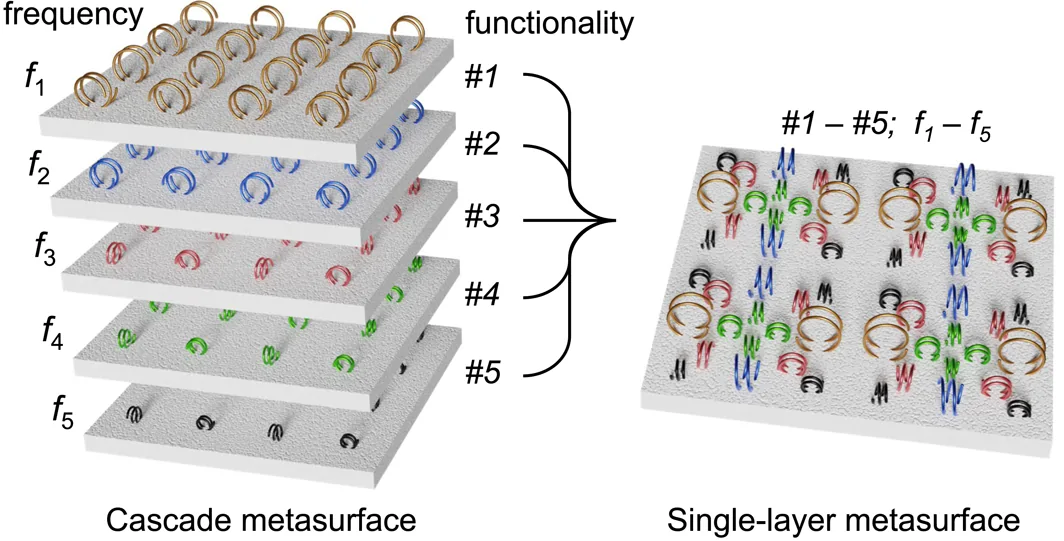
A schematic illustration of a transition from a cascade to a single‐layer metasurface remaining the same functionalities and operational frequencies.

By carefully engineering the electric, magnetic, and magnetoelectric responses of individual helical resonators and exploiting their frequency selectivity, we show that the proposed single‐layer metasurface reproduces the full functionality of its cascaded counterpart with only minor performance deviations. Numerical simulations confirm high efficiency, polarization robustness, and angular stability across all operational bands. This cascade‐to‐single‐layer transformation provides a general strategy for reducing metasurface complexity without sacrificing performance, offering a scalable route toward ultracompact multifunctional electromagnetic devices.

## 2 Principle and Design

Here, we focus on the design of highly efficient multifunctional multispectral metasurfaces based on helical resonators with optimized electromagnetic responses. The considered helical resonators are inherently chiral, and their electromagnetic response is governed by the simultaneous excitation of electric and magnetic dipoles [37, 38]. Typically, helical meta‐atoms employed for efficient polarization conversion rely on their intrinsic chirality [39, 40, 41], linear polarization rotation [7, 42], and perfect absorption [43, 44].

We employ three‐dimensional smooth metallic helices as meta‐atoms, whose geometric parameters are individually optimized to achieve balanced axial electric dipole (${\text{ED}}_{x}$) and magnetic dipole (${\text{MD}}_{x}$) responses at distinct resonance frequencies. The strength of polarization rotation and conversion is maximized when the induced electric and magnetic dipole moments satisfy the electromagnetic balance condition ${\text{ED}}_{x}$ = ${\text{MD}}_{x}$, which corresponds to the formation of a Huygens' dipole pair [45, 46]. This balance condition can be achieved through careful optimization of the helix geometry, allowing precise control of the induced dipole amplitudes at the desired operating frequency. Thus, by tuning the helix radius, height, and wire diameter, each helical resonator is designed to operate efficiently at a specific frequency while remaining weakly responsive outside its resonance band. This approach enables the realization of individual helix‐based metasurfaces supporting five distinct electromagnetic functionalities, each associated with a different operating frequency:

- 4 GHz: Magnetic Mirror Selective Surface (MMSS);
- 5 GHz: Circular Polarization Selective Surface (CPSS);
- 6 GHz: Reflective Polarization Rotator (RPR);
- 7 GHz: Transmissive Polarization Rotator (TPR);
- 8 GHz: Perfect Absorber (PA).

Figure 2 shows the frequency dependence of the normalized scattering cross‐sections associated with the axial ${ED}_{x}$ and ${MD}_{x}$ moments of the optimized helical resonators. The helix axis is aligned along the x direction in all cases. All numerical simulations presented in this work were performed using the finite‐element solver COMSOL Multiphysics. Figure 2a–d correspond to copper helices designed to operate at 4, 5, 6, and 7 GHz, respectively. For each design, the geometric parameters of the helix, such as the radius $r_{i}$, pitch $h_{i}$, and wire diameter $d_{0}$, are optimized such that the ${ED}_{x}$ and ${MD}_{x}$ scattering cross‐sections overlap at the resonance frequency, indicating balanced electric and magnetic dipole responses.

**FIGURE 2:**
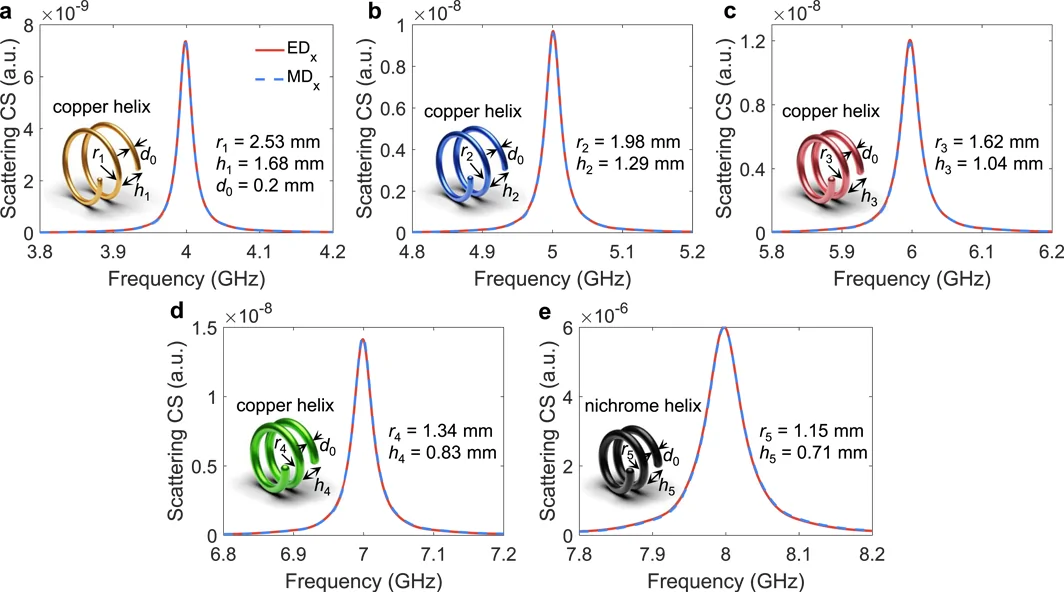
Scattering cross‐section of axial electric and magnetic dipole moments of optimized helices around frequencies (a) 4 GHz, (b) 5 GHz, (c) 6 GHz, (d) 7 GHz, and (e) 8 GHz, respectively. Optimized structural parameters are indicated in the inset of each figure.

Figure 2e shows the corresponding scattering cross‐sections of axial dipolar moments for a nichrome helix designed to operate at 8 GHz. In this case, the higher material losses of nichrome are exploited to realize efficient absorption while maintaining the balance between ${ED}_{x}$ and ${MD}_{x}$. It should be noted that other lossy metals, such as titanium, can be used as suitable alternatives, as the conductivities of these metals are of the same order of magnitude. The electromagnetic balance of the helices can also be described in terms of their polarizabilities, as shown in Supporting Information S1: Figure S1, providing additional insight into the underlying electric‐magnetic coupling mechanisms. To verify that the proposed helices predominantly excite only diagonal components of the electric and magnetic dipole moments, all components of the normalized scattering cross‐sections were evaluated, as shown in Supporting Information S1: Figure S2. These results demonstrate that by appropriately tailoring the helix geometry and material composition, balanced dipolar responses can be achieved at distinct target frequencies, enabling the realization of individual metasurface functionalities across the 4–8 GHz range.

## 3 Cascade Metasurface

We first examine the structural design and electromagnetic performance of the proposed multifunctional cascaded metasurface. Figure 3 presents the structure and electromagnetic performance of the proposed five‐layer cascaded metasurface, in which each layer is designed to realize a distinct functionality at a predefined operating frequency.

**FIGURE 3:**
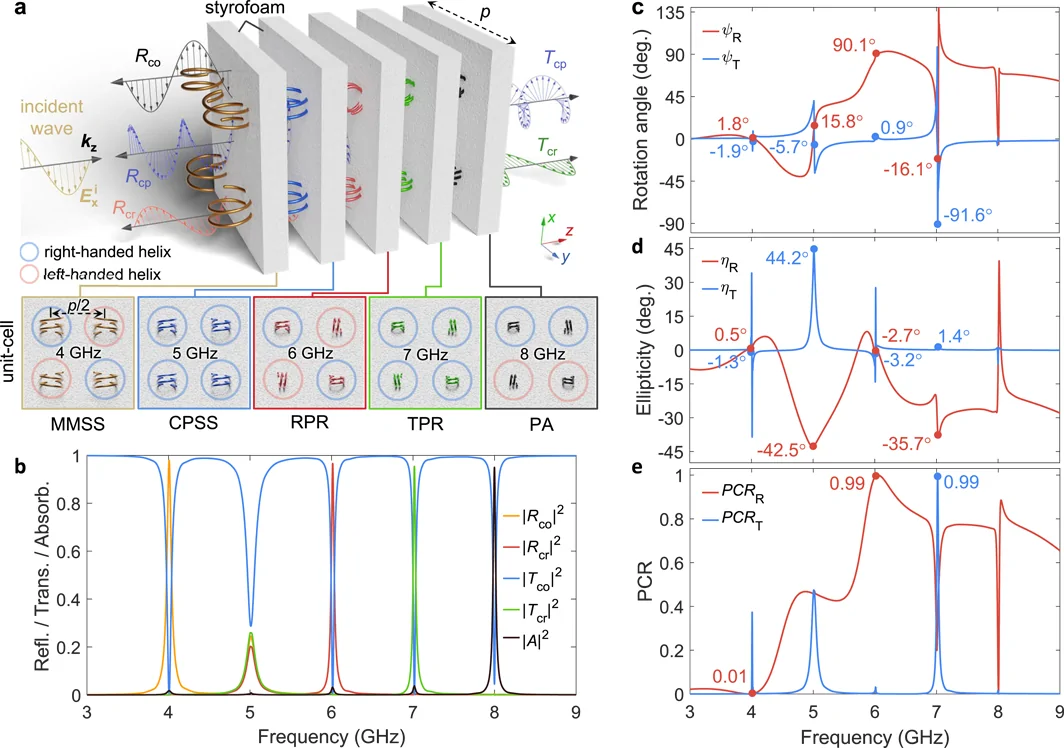
Cascade metasurface and its polarization characteristics. (a) Schematic image of cascade metasurface consisting of balanced helices depicted with the function of each layer at the corresponding frequency. (b) Co‐ and cross‐polarized reflection, transmission and absorption spectra at normal incidence. (c) Rotation angle, (d) ellipticity, and (e) polarization conversion ratio of reflected and transmitted waves of the proposed cascade metasurface. The blue and red circles indicate the arrangement of right‐ and left‐handed helices.

Figure 3a schematically illustrates the unit cell of the cascade metasurface. The metasurface consists of five spatially separated single‐layer metasurfaces embedded in a styrofoam host medium and stacked along the propagation direction of the x‐polarized incident plane wave. The styrofoam exhibits a relative permittivity of $\varepsilon_{r}\approx 1.03$ in the operating frequency range, making it transparent to RF waves and serving mainly as a mechanical support for the helical inclusions without influence on the electromagnetic response. Each layer comprises an array of right‐handed and/or left‐handed helical resonators arranged within a square unit cell of period p=22 mm, with an inter‐helix spacing of p/2. The layers are designed to operate independently at 4, 5, 6, 7, and 8 GHz, corresponding to the magnetic mirror selective surface (MMSS), circular polarization selective surface (CPSS), reflective polarization rotator (RPR), transmissive polarization rotator (TPR), and perfect absorber (PA), respectively. The chosen frequency spacing (1 GHz interval between 4 and 8 GHz) is not dictated by a fundamental limitation of the resonance bandwidth. It is chosen for clarity of presentation and to avoid cross‐talk interaction between different functionalities. It should be noted that the metasurface operating at 8 GHz has a period exceeding λ/2. However, its operation relies on the engineered response of individual unit cells rather than a homogenized approximation. Although a larger period may support higher‐order Floquet modes, their excitation is weak due to the geometry and normal incidence. As a result, no significant parasitic diffraction occurs, and the metasurface maintains its functionality. By analyzing the real and imaginary parts of the induced electric and magnetic dipole moments (see Supporting Information S1: Figure S3), the orientation and handedness of the helices in each layer are selected to achieve the targeted polarization response, in accordance with the required dipole orientation and excitation within the metasurface unit cell [44, 47, 48, 49].

Figure 3b shows the frequency‐dependent normalized co‐ and cross‐polarized reflection ($|R_{\text{co}}{|}^{2}$, $|R_{\text{cr}}{|}^{2}$), transmission ($|T_{\text{co}}{|}^{2}$, $|T_{\text{cr}}{|}^{2}$), and absorption $\left(|A{|}^{2}\right)$ coefficients at normal incidence. Well‐defined resonant features are observed at the five design frequencies, confirming that each layer performs its intended function with high spectral selectivity. At 4 GHz, the MMSS exhibits near‐unity co‐polarized reflection with a reflection phase close to 0°, while transmission and absorption remain negligible, which is characteristic of magnetic mirror behavior. At 5 GHz, the CPSS shows balanced circularly polarized reflection and transmission. The RPR and TPR layers at 6 and 7 GHz exhibit strong cross‐polarized reflection and transmission around 97%, respectively, while the PA at 8 GHz achieves above 96% absorption.

Figure 3c presents the polarization rotation angle of the reflected and transmitted waves as a function of frequency. Pronounced rotation is observed only at the resonance frequencies associated with the RPR and TPR layers, where the rotation angles approach ±90°, indicating efficient polarization rotation. At other frequencies, the rotation angle remains close to zero, confirming minimal cross‐coupling between the different functionalities. Figure 3d shows the corresponding ellipticity of the reflected and transmitted waves. The CPSS layer at 5 GHz introduces significant ellipticity, approaching ±45°, which confirms efficient conversion from linear to circular polarization. In contrast, the MMSS, RPR, TPR, and PA layers exhibit near‐zero ellipticity at their respective operating frequencies, preserving linear polarization where required. Figure 3e displays the polarization conversion ratio (PCR) for reflection and transmission of the cascade metasurface. High PCR values close to unity are achieved at the resonance frequencies of the RPR and TPR layers, confirming efficient polarization conversion, while negligible PCR is observed outside the target bands.

According to numerical simulations (see Supporting Information S1: Figure S4), the optimal unit‐cell period generally differs for each functional layer in order to maximize the electromagnetic response at its corresponding resonance frequency. For simplicity in the numerical simulations, a uniform unit‐cell period p was adopted for all layers in the cascaded metasurface shown in Figure 3a. The influence of inter‐element spacing on the electromagnetic response is further analyzed in Supporting Information S1: Figure S5. The results show that increasing the inter‐helical distance primarily leads to a systematic shift of the resonance frequency, while the peak amplitude of the electromagnetic response remains nearly unchanged. This behavior is observed for all functional layers except the perfect absorber. Efficient absorption requires a sufficiently strong near‐field coupling between the helices; therefore, placing the absorbing helices too close to the center or too close to the unit‐cell boundaries results in a reduction of the absorption amplitude.

The polarization response of the cascaded metasurface is examined in Supporting Information S1: Figure S6. The results confirm that the metasurface is polarization‐insensitive under linearly polarized excitation, as the normalized reflection, transmission, and absorption coefficients remain identical for both x‐ and y‐polarized incident waves at the corresponding resonance frequencies. The polarization characteristics of the metasurfaces also remain consistent performance for both orthogonal linear polarizations of the incident wave (see Supporting Information S1: Figure S7). This polarization‐independent behavior is preserved across all functional layers, validating the robustness of the proposed design.

The proposed cascaded metasurface, based on balanced double‐turn helical resonators, enables independent and high‐efficiency realization of multiple electromagnetic functionalities with minimal mutual interference between individual layers. Therefore, this multilayer architecture can be systematically collapsed into a single‐layer metasurface owing to the strong frequency selectivity of the individual metasurfaces and their high transparency outside the resonance bands.

## 4 Single‐Layer Metasurface

Here, we propose the design of a single‐layer multifunctional multispectral metasurface, whose design and performance are summarized in Figure 4. Figure 4a shows a schematic illustration of the single‐layer supercell with period p=22 mm, in which all five functionalities are integrated within a single planar structure. Each helical resonator is color‐coded to indicate its associated functionality, corresponding to operating frequencies of 4, 5, 6, 7, and 8 GHz. For most functionalities operating at lower frequencies, only two helices are required, enabling a compact supercell that accommodates all five devices simultaneously.

**FIGURE 4:**
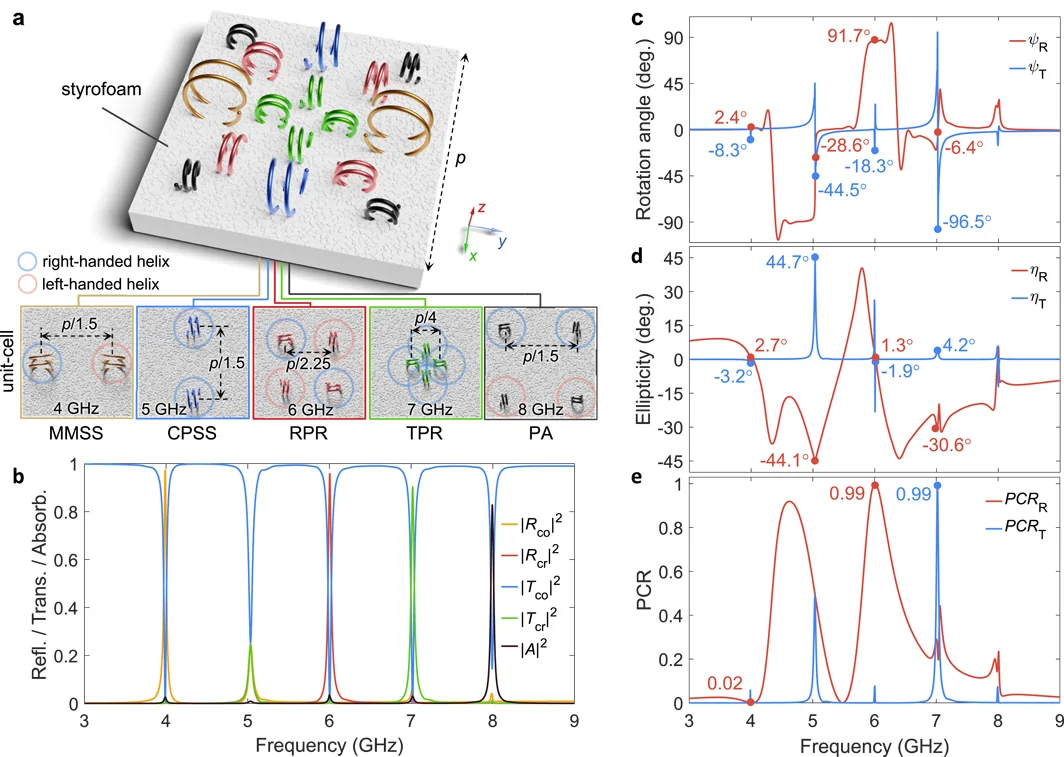
Single‐layer metasurface and its polarization characteristics. (a) Schematic image of helix‐based metasurface depicted with the function of each layer at the corresponding frequency. (b) Co‐ and cross‐polarized reflection, transmission and absorption spectra at normal incidence. (c) Rotation angle, (d) ellipticity, and (e) PCR for reflection and transmission, respectively.

As in the cascaded configuration, right‐handed helices are indicated by blue circles, while left‐handed helices are marked by red circles. The spatial arrangement and inter‐helical spacing of each functional subset are individually optimized to ensure minimal mutual coupling and efficient excitation of the corresponding resonant response (see Supporting Information S1: Figure S8). Despite the collapse of the multilayer architecture into a single planar layer, the supercell preserves the functional separation between different metasurface operations through spatial multiplexing of frequency‐selective helices.

The frequency‐dependent normalized co‐ and cross‐polarized reflection, transmission, and absorption coefficients of the single‐layer metasurface are presented in Figure 4b. Distinct resonant features are observed at the designated operating frequencies, demonstrating that all five functionalities are successfully reproduced within the single‐layer configuration. Overall, the electromagnetic response closely matches that of the cascaded metasurface shown in Figure 3b. Minor deviations are observed for the TPR and PA: the cross‐polarized transmission coefficient of the TPR reaches approximately 0.9 at 7 GHz, and the absorption coefficient of the PA does not exceed 0.85 at 8 GHz. These deviations can be attributed to the spatial placement of the helices associated with the TPR and PA functionalities located near the center and the boundary of the unit cell, respectively, which results in slightly reduced performance compared to the layer‐by‐layer cascaded configuration. Also, the resonant peaks in Figure 4b appear narrower compared to those in the cascaded configuration in Figure 3b. This behavior is mainly due to the reduced number of resonant inclusions in the unit cell. In the cascaded design, four helical resonators produce a broader and more symmetric response through resonance superposition. In contrast, the single‐layer metasurfaces (MMSS and CPSS) include only two helices, leading to more isolated resonances with higher Q‐factor, increased asymmetry, and narrower bandwidth. Moreover, the reduced number of inclusions makes the response more sensitive to inter‐element spacing and mutual coupling.

The polarization properties of the single‐layer metasurface are further characterized in Figure 4c–e. The obtained results closely follow those of the cascaded metasurface, confirming effective polarization manipulation for the relevant functionalities. Only minor differences are observed in polarization rotation angles, with deviations not exceeding 9°, which are negligible for practical applications. Together, these results demonstrate that the proposed single‐layer metasurface successfully replicates the functionality of the cascaded design while significantly reducing structural thickness and fabrication overhead.

Further, we evaluate the angular response of the proposed metasurfaces under oblique incidence of electromagnetic waves. Figure 5a,b present the angle‐ and frequency‐dependent normalized reflection, transmission, and absorption coefficients for the cascaded and single‐layer metasurfaces, respectively. For the cascaded metasurface, the MMSS exhibits the highest angular stability, maintaining large normalized cross‐polarized reflection amplitudes up to incidence angles of approximately 70°. In contrast, the remaining functionalities of the CPSS, RPR, TPR, and PA retain efficient operation only for incidence angles below ∼50°. In particular, the CPSS layer maintains nearly equal values of $|R_{\text{cp}}{|}^{2}$ and $|T_{\text{cp}}{|}^{2}$ close to 0.5, corresponding to balanced splitting of the incident linearly polarized wave into reflected and transmitted circularly polarized waves. Figure 5b shows the corresponding angular response of the single‐layer metasurface where all five functionalities remain clearly observable for incidence angles up to approximately 40°–45°. Although this angular tolerance is reduced compared to the cascaded configuration, it remains sufficient for most practical applications.

**FIGURE 5:**
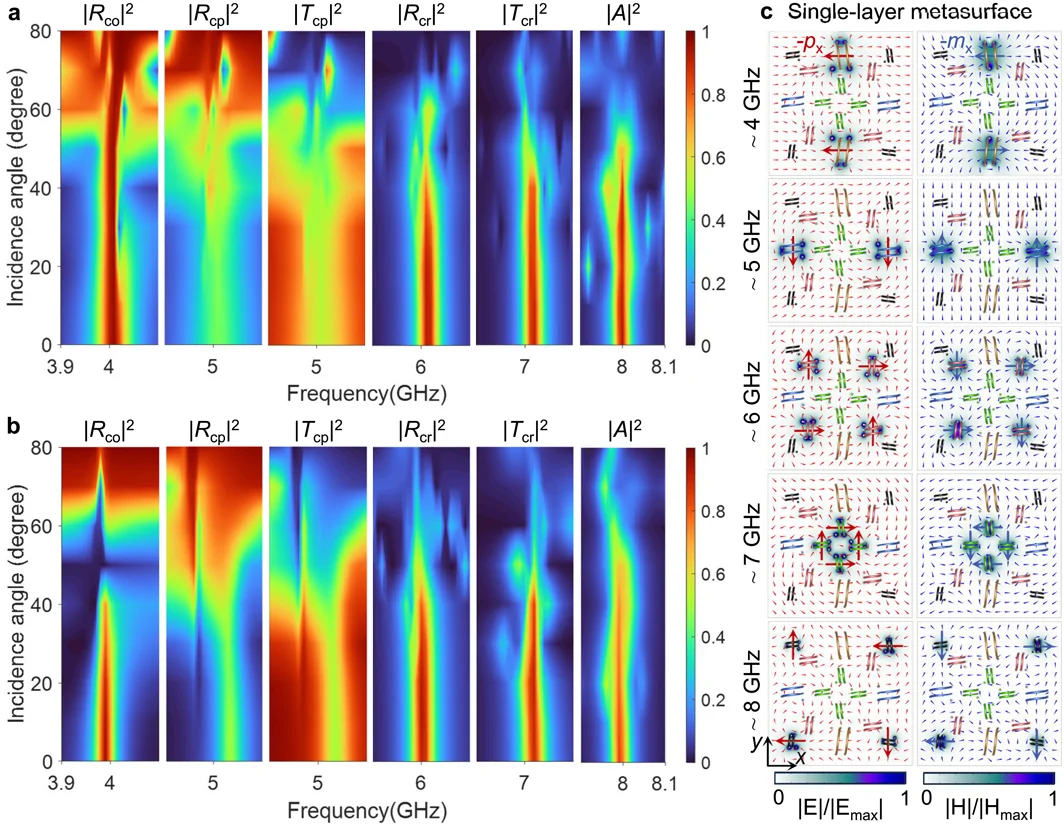
(a, b) Co‐/cross‐/circular‐polarized reflection, transmission, and absorption spectra as a function of the incidence angle of the cascade and single‐layer metasurface. (c, d) Distribution of normalized electric and magnetic fields at the resonance frequency of each layer of the cascade and single‐layer metasurface. Red and blue arrows show the direction of electric and magnetic dipole moments.

Figure 5c provides further physical insight into the operation of the single‐layer metasurface by showing the spatial distributions of the normalized electric and magnetic fields at the resonance frequencies of each functionality, together with the corresponding vector distributions of the induced electric and magnetic dipole moments within the unit cell. Large red and blue arrows indicate the orientations of the dominant electric and magnetic dipole moments excited in the helices. At each resonance frequency, only the helices associated with the corresponding functionality are strongly excited, while the remaining helices exhibit negligible response.

These results confirm that the proposed single‐layer metasurface preserves selective excitation of frequency‐dependent functionalities even under oblique incidence. Additional field distributions in transverse cross‐sections for both the cascaded and single‐layer metasurfaces at different operating frequencies are provided in Supporting Information S1: Figure S9.

In summary, the general design approach starts with selecting meta‐atoms capable of supporting the required electromagnetic responses, for example, elements that can realize balanced electric and magnetic (Huygens‐type) resonances. First, a single functionality is designed at the lowest target frequency by arranging the meta‐atoms within a unit cell, typically in a symmetric configuration to ensure the desired response. The spectral behavior of this unit cell is then analyzed to identify its transparency region, that is, the frequency range beyond the main resonance where the structure exhibits minimal interaction with the incident wave. A second functionality is subsequently designed at a higher frequency, ensuring that it lies within the same transparency band of the first design. The corresponding meta‐atoms are then introduced into the same unit cell, with careful adjustment of their geometry and spacing to control mutual coupling. This process is repeated for additional functionalities, each time ensuring minimal cross‐talk by proper spectral separation and spatial arrangement of the inclusions. Finally, the entire structure can be scaled to other frequency ranges by proportionally adjusting the geometrical parameters with respect to the wavelength, while accounting for material dispersion and losses.

## 5 Versatility of Single‐Layer Metasurfaces

Next, we demonstrate the versatility of the proposed single‐layer metasurface design by reconfiguring each of the five core functionalities to operate at multiple frequency bands. By appropriately optimizing the geometric parameters of the helices and their spatial arrangement within the unit cell, each functionality can be independently realized at arbitrary target frequencies without altering the overall design framework. The supercell designs and corresponding electromagnetic responses of these multifrequency implementations are summarized in Figure 6.

**FIGURE 6:**
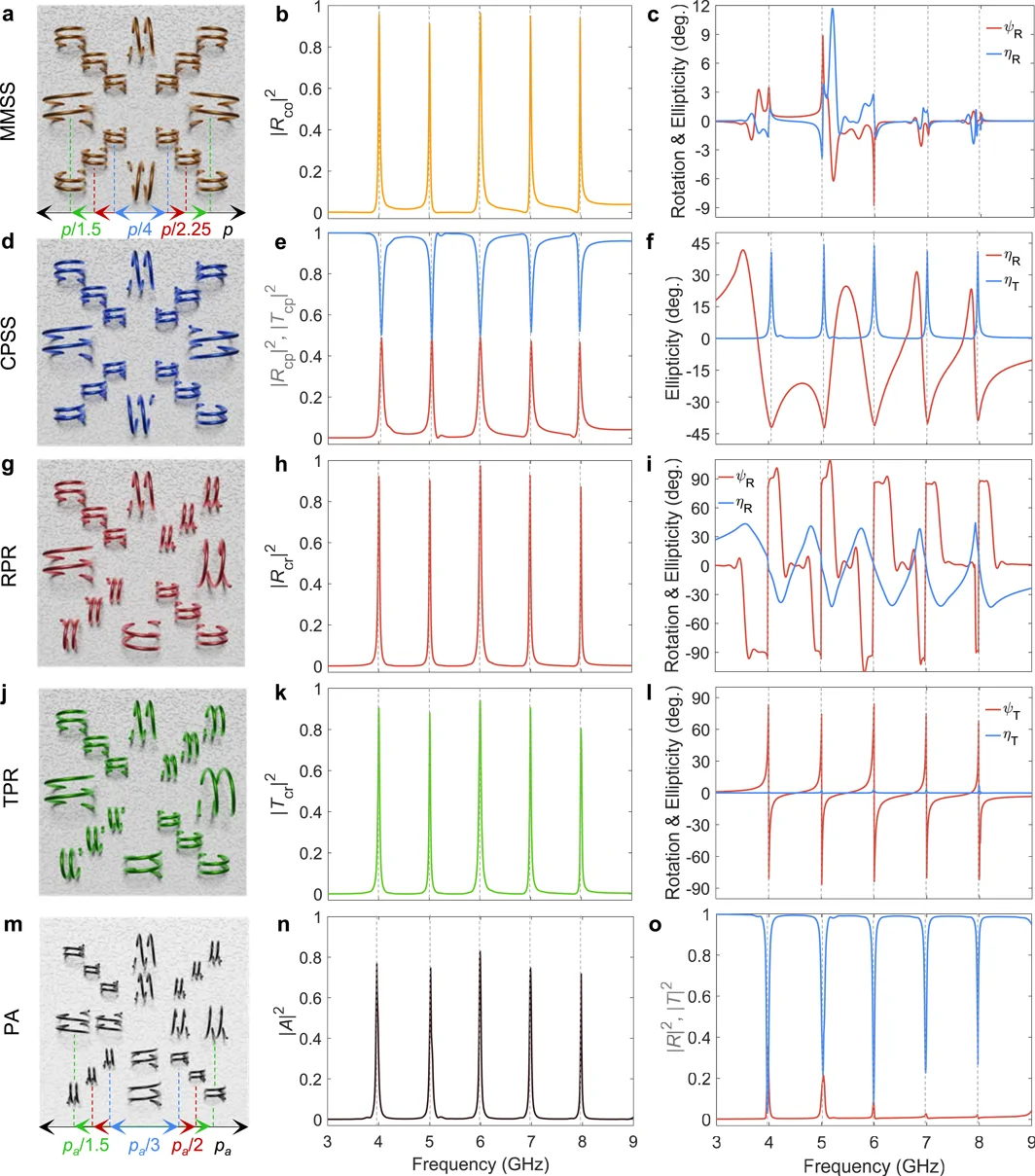
(a–c) Multifrequency design of MMSS and its co‐reflection spectra along with rotation and ellipticity. (d–f) CPSS design with circular‐polarized reflection, transmission spectra and ellipticity. (g–i) CPR design with its cross‐reflection spectra, rotation and ellipticity. (j–l) CPT design with its cross‐transmission spectra, rotation and ellipticity. (m–o) PA design and its absorption, transmission, and reflection spectra.

Figure 6a shows the corresponding unit‐cell of single‐layer multifrequency MMSS, while Figure 6b displays the co‐polarized reflection spectra. At each designed resonance frequency, the reflection coefficient $|R_{\text{co}}{|}^{2}$ reaches values between 0.90 and 0.96. The associated polarization rotation angles and ellipticity, shown in Figure 6c, remain close to zero across all operating frequencies, confirming preservation of linear polarization behavior.

The multifrequency CPSS is shown in Figure 6d–f. As seen in Figure 6e, both the circularly polarized reflection and transmission coefficients, $|R_{\text{cp}}{|}^{2}$ and $|T_{\text{cp}}{|}^{2}$, approach values of approximately 0.5 at each resonance frequency, indicating equal splitting of the incident wave. The ellipticity plots in Figure 6f show values close to ±45°, confirming efficient linear‐to‐circular polarization conversion across all frequency bands.

Figure 6g–i illustrate the multifrequency operation of the RPR. The cross‐polarized reflection coefficient $|R_{\text{cr}}{|}^{2}$ reaches values between 0.90 and 0.98 at the designated resonance frequencies, as shown in Figure 6h. The ellipticity remains close to zero, while the polarization rotation angles shown in Figure 6i approach 89° on average, confirming effective polarization rotation of the reflected wave.

The TPR is demonstrated in Figure 6j–l. The results show high cross‐polarized transmission at each target frequency, with polarization rotation and ellipticity characteristics closely matching those of the reflective counterpart, confirming robust polarization rotation in transmission over multiple frequency bands.

Finally, the multifrequency implementation of the PA is presented in Figure 6m–o. In contrast to the other functionalities, realization of efficient absorption requires a minimum of four helices per unit cell, leading to a distinct spatial configuration with specifically optimized inter‐helical spacings. As shown in Figure 6n, the absorption coefficient $|A{|}^{2}$ reaches values between 0.72 and 0.82 at the resonance frequencies. The slightly reduced absorption performance compared to single‐frequency designs is attributed to enhanced mutual coupling between closely packed helices, which perturbs the balance between electric and magnetic dipole moments.

Alternative supercell designs with different sequences and spatial combinations of the functional elements are presented in Supporting Information S1: Figures S10 and S11. These results demonstrate that the proposed single‐layer metasurface architecture preserves its electromagnetic functionality under various arrangements, confirming the flexibility of the design and the robustness of the individual frequency‐selective responses. Overall, the results in Figure 6 confirm that the proposed single‐layer metasurface provides a flexible and scalable platform for multifunctional electromagnetic devices, enabling independent reconfiguration of each functionality across multiple frequency bands while maintaining robust performance.

Importantly, the demonstrated flexibility is not limited to a specific arrangement or frequency assignment but represents a general design paradigm. By decoupling the spatial placement of the meta‐atoms from their spectral functionality, the proposed single‐layer metasurface allows independent optimization of geometry, operating frequency, and electromagnetic response within a single planar structure. This modularity enables straightforward adaptation of the metasurface to different application requirements, such as shifting frequency bands, prioritizing specific functionalities, or modifying the number of supported operations, without altering the underlying design concept. As a result, the proposed architecture provides a versatile platform for the development of compact, application‐tailored multifunctional metasurfaces with minimal redesign effort.

The proposed single‐layer multifunctional multispectral metasurface represents a distinct class of passive designs capable of realizing different electromagnetic functionalities (reflection, transmission, and absorption) at separate frequencies within a single unit cell. Unlike conventional approaches, which typically operate within a single regime or repeat the same functionality across multiple resonances, the present design integrates diverse responses without the need for multilayer stacking or active tuning. Moreover, the cascade‐to‐single‐layer transformation preserves the targeted functionalities with minimal performance degradation, demonstrating an efficient strategy for compressing complex electromagnetic responses into a unified architecture.

Towards practical realization of the proposed metasurfaces, consisting of copper helices embedded in a styrofoam, can be fabricated with high accuracy using established techniques such as precise wire pre‐forming for helical or curved elements, as demonstrated in previous works on wire‐based metasurfaces [39, 44, 47]. These studies indicate that moderate fabrication imperfections may lead to slight resonance shifts and minor efficiency degradation, while the overall functionality such as polarization control, absorption, or transmission, remains largely preserved due to the reliance on collective electromagnetic responses rather than sharp high‐Q resonances. In addition, modern metal additive manufacturing techniques, such as selective laser melting (SLM) [50] and direct metal laser sintering (DMLS) [51], provide a viable route for realizing complex geometries with sub‐millimeter precision. Therefore, experimental verification at microwave frequencies is feasible using standard fabrication and measurement approaches, further supporting the practical relevance of the proposed design.

## 6 Conclusion

In summary, we have demonstrated a systematic strategy for realizing highly efficient multifunctional metasurfaces by collapsing a cascaded multilayer architecture into an ultrathin single‐layer implementation while largely preserving electromagnetic performance. Starting from a five‐layer cascaded metasurface that supports magnetic‐mirror operation, circular‐polarization selectivity, reflective and transmissive polarization rotation, and perfect absorption at distinct frequencies, we established explicit design principles based on frequency‐selective helical resonators with balanced axial electric‐ and magnetic‐dipole responses. The cascaded metasurface exhibits high efficiency, strong polarization control, and robust angular performance, providing a benchmark for multifunctional operation. By spatially multiplexing optimized helices within a single planar supercell, the single‐layer metasurface successfully reproduces all five functionalities with comparable spectral selectivity, polarization response, and field localization, despite minor deviations at higher frequencies.

Beyond reproducing the cascaded response, the proposed single‐layer metasurface demonstrates substantial flexibility and generality. The design supports polarization‐insensitive operation under linearly polarized illumination, maintains stable functionality under oblique incidence up to moderate angles, and enables selective excitation of individual functions at their designated resonance frequencies, as corroborated by field distributions and dipole‐moment analyses. Moreover, the architecture permits straightforward reconfiguration of operating frequencies and spatial layouts, and it can realize multiple frequency bands for a given functionality within the same planar framework. Collectively, these results establish the proposed single‐layer metasurface as a compact, scalable, and fabrication‐friendly platform for advanced electromagnetic wave manipulation, offering a practical route toward integrated multifunctional devices for communication, sensing, and polarization‐control applications.

## Author Contributions

Ih.F. conceived the idea and performed the full‐wave simulations. Ih.F. and Iv.F. drafted the manuscript, contributing to its review and revision.

## Conflicts of Interest

The authors declare no conflicts of interest.

## Supporting information


Supporting Information S1

## Data Availability

The data that support the findings of this study are available from the corresponding author upon reasonable request.
