# Supplementary material for: Cascade‐To‐Single Layer Transformation of Multifunctional Multispectral Metasurfaces
Source: Nanophotonics. 2026 May 11;15(9):e70132. doi: 10.1002/nap2.70132 (PMC13642071; doi:10.1002/nap2.70132)
Supplement: Supplementary file 1 [file NAP2-15-e70132-s001.pdf]

Supplementary material for the paper

"Cascade-to-Single Layer Transformation of Multifunctional Multispectral Metasurfaces"

I. Faniayeu and I. Fanyaev

Contents

1 Balanced polarizabilities of the helices 2

2 Multipolar decomposition of the helices 3

3 Parametrization and optimization of metasurfaces unit cells 6

4 Polarization-independence of the single-layer unit-cell metasurface 11

5 Robustness of single-layer multifunctional multispectral metasurface 13

1 Balanced polarizabilities of the helices

Since the targeted metasurface functionality is realized using helical resonators, its electromagnetic response can be engineered through the individual polarizabilities of the constituent meta-atoms. The considered helical resonators are intrinsically bianisotropic particles, characterized by four fundamental polarizabilities: electric (α_{ee}), magnetic (α_{mm}), electromagnetic (α_{em}), and magnetoelectric (α_{me}). These polarizabilities describe the linear relationship between the locally applied electromagnetic fields and the induced electric and magnetic dipole moments of the resonators.

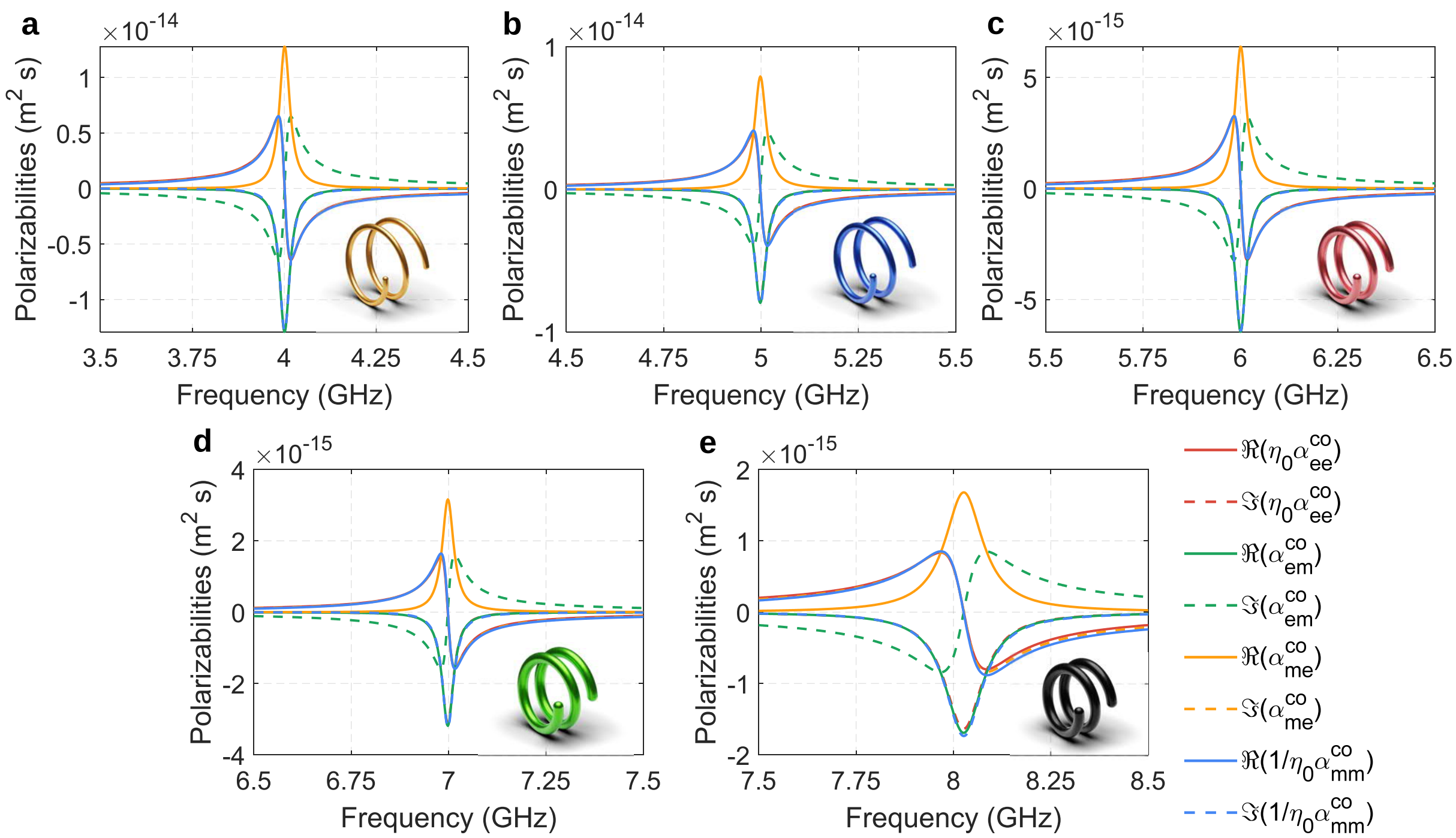

Figure S1: Individual polarizabilities of the helices for target resonance frequency of (a) 4 GHz, (b) 5 GHz, (c) 6 GHz, (d) 7 GHz, and (e) 8 GHz, respectively.

To achieve the desired metasurface performance, a proper balance between these polarizabilities is required. In particular, for linear polarized plane waves, the polarizability balance condition can be expressed in terms of the individual polarizabilities as [S1, S2]

$$\eta_0 \alpha_{ee}^{\rm co} = 1/\eta_0 \alpha_{mm}^{\rm co}, \quad \alpha_{em}^{\rm co} = -\alpha_{me}^{\rm co}, \tag{1}$$

where η_0 is the free-space wave impedance. While all the cross-polarizabilities of helices are equal to zero. Using a general retrieval approach applicable to particles of arbitrary geometry [S1], we calculated the dominant co-polarized polarizabilities of the helical resonators. Fig S1 presents the frequency-dependent co-polarized (axial) polarizabilities of the helices optimized for the target operating resonant frequencies. As shown, the polarizability balance condition given by Eq. (1) is satisfied by appropriately tailoring the geometric parameters of the helices, in particular their height and radius, enabling the realization of the desired metasurface functionalities.

2 Multipolar decomposition of the helices

The total scattering cross section $C_{\rm sca}^{\rm total}$ of helices can be directly expressed as the sum of the contributions from the individual electric and magnetic dipole moments as [S3]

$$C_{\rm sca}^{\rm total} = C_{\rm sca}^{p} + C_{\rm sca}^{m} + \cdots = \frac{k^4}{6\pi\varepsilon_0^2 |E_{\rm inc}|^2} \left[\sum_i \left(|p_i|^2 + \frac{|m_i|^2}{c^2} \right) + \cdots \right], \tag{2}$$

where p_i and m_i are the electric and magnetic dipole moments, $i = \{\text{x, y, z}\}$, $|E_{\rm inc}|$ denotes the electric field amplitude of the incident plane wave, k is the wavenumber, and ε_0 is the permittivity of free space.

According to the multipole decomposition within the long-wavelength approximation [S4], the dominant contributions arise from the electric dipole (ED) and magnetic dipole (MD) moments, described as follows:

$$\text{ED}_i: \quad p_i \approx -\frac{1}{i\omega}\left\{\int \mathrm{d}^3\mathbf{r}\, J_{\omega,i} + \frac{k^2}{10}\int \mathrm{d}^3\mathbf{r}\left[(\mathbf{r}\cdot\mathbf{J}_\omega)\, r_i - 2r^2 J_{\omega,i}\right]\right\}, \tag{3}$$

$$\text{MD}_i: \quad m_i \approx \frac{1}{2}\int \mathrm{d}^3\mathbf{r}\; (\mathbf{r}\times\mathbf{J}_\omega)_i\,, \tag{4}$$

where J_ω is induced electric current, ω is angular frequency. Based on the above expressions (3)-(4), the electric and magnetic dipole moments associated with the proposed helical resonators were evaluated.

Based on Eq. (2), the normalized scattering cross-sections of the electric and magnetic dipole moments along the x, y, and z directions are calculated and presented in Fig. S2. At the resonant frequencies, the axial components ED_x and MD_x attain equal values. Additionally, a nonzero value is observed for the electric dipole moment component ED_z.

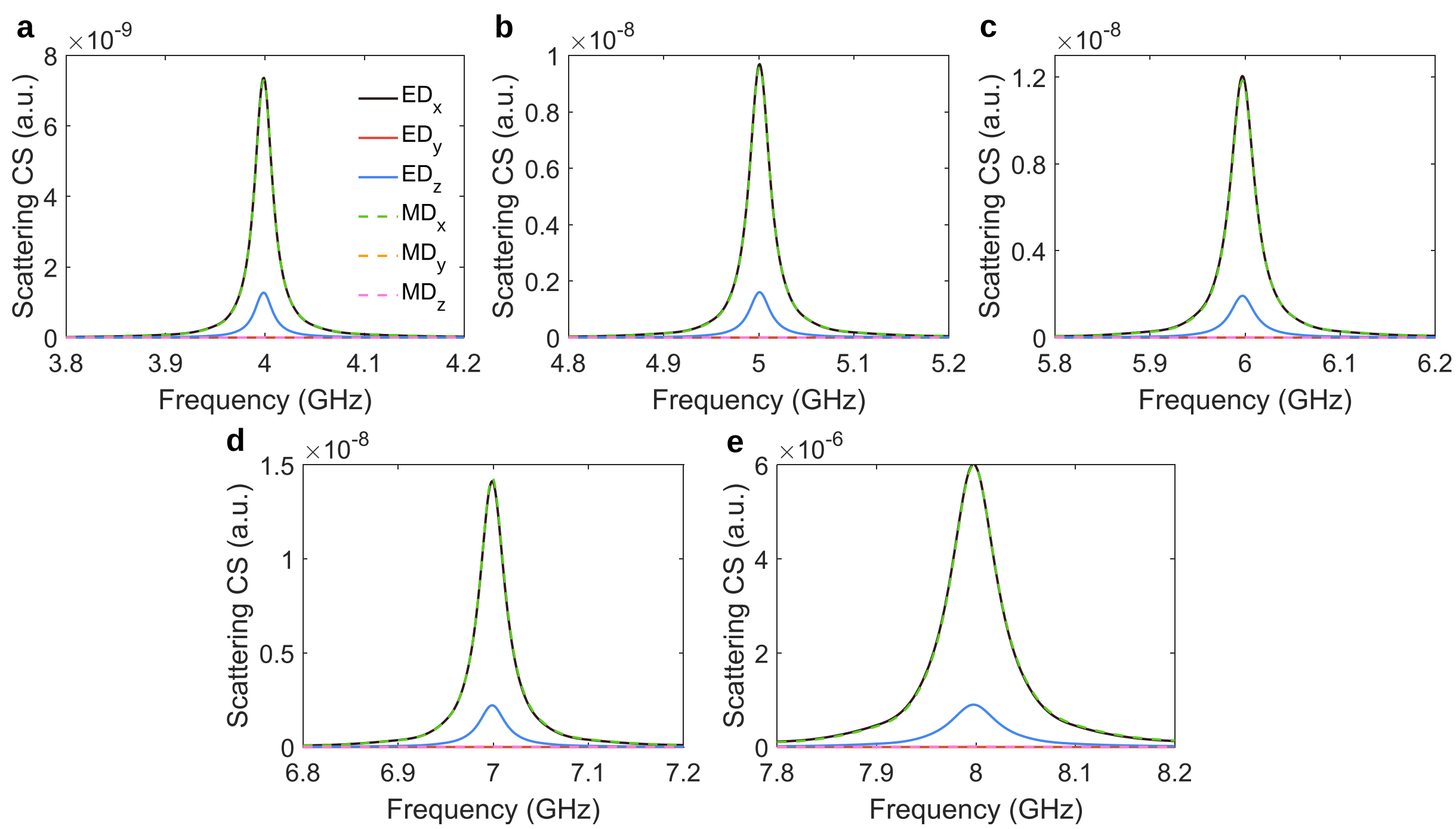

Figure S2: The components of electric and magnetic dipole moments of proposed helices at resonances: (a) 4 GHz, (b) 5 GHz, (c) 6 GHz, (d) 7 GHz, and (e) 8 GHz, respectively.

Fig. S3 presents the real and imaginary parts of the electric and magnetic dipole moments as functions of frequency for different orientations of the axes of right- and left-handed helices at the resonance frequency of 4 GHz. As described in Ref. [S5], a systematic analysis of the induced dipole moments associated with differently oriented right- and left-handed helices allows determination of the optimal unit-cell arrangement required to realize desired MMSS, CPSS, RPR, TPR and PA metasurfaces.

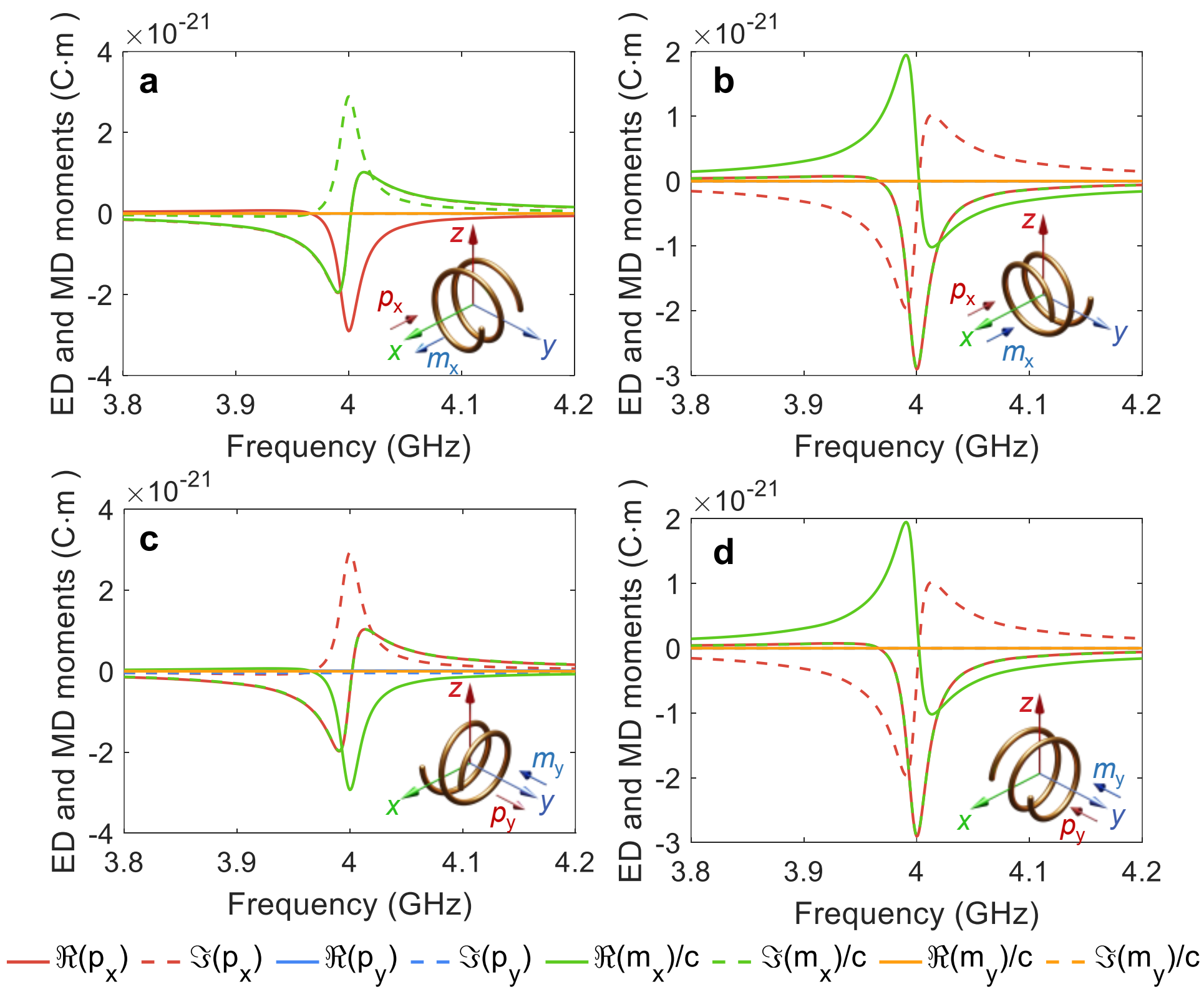

Figure S3: The real and imaginary parts of the electric and magnetic dipole moments are shown for different helix orientations: (a) a right-handed helix aligned along the x-axis, (b) a left-handed helix aligned along the x-axis, (c) a right-handed helix aligned along the y-axis, and (d) a left-handed helix aligned along the y-axis. The orientation of each helix and the corresponding dominant induced dipole moments are indicated in the inset of each panel.

3 Parametrization and optimization of metasurfaces unit cells

Fig. S4 illustrates the influence of the unit-cell period p on the electromagnetic response of the individual functional metasurfaces. Panels (a)–(e) correspond to the five targeted functionalities, namely co-polarized reflection (MMSS), circular polarization splitting (CPSS), cross-polarized reflection (RPR), cross-polarized transmission (TPR), and absorption (PA), respectively. For each functionality, the resonant response remains well defined while the resonance frequency slightly shifts with increasing unit-cell period.

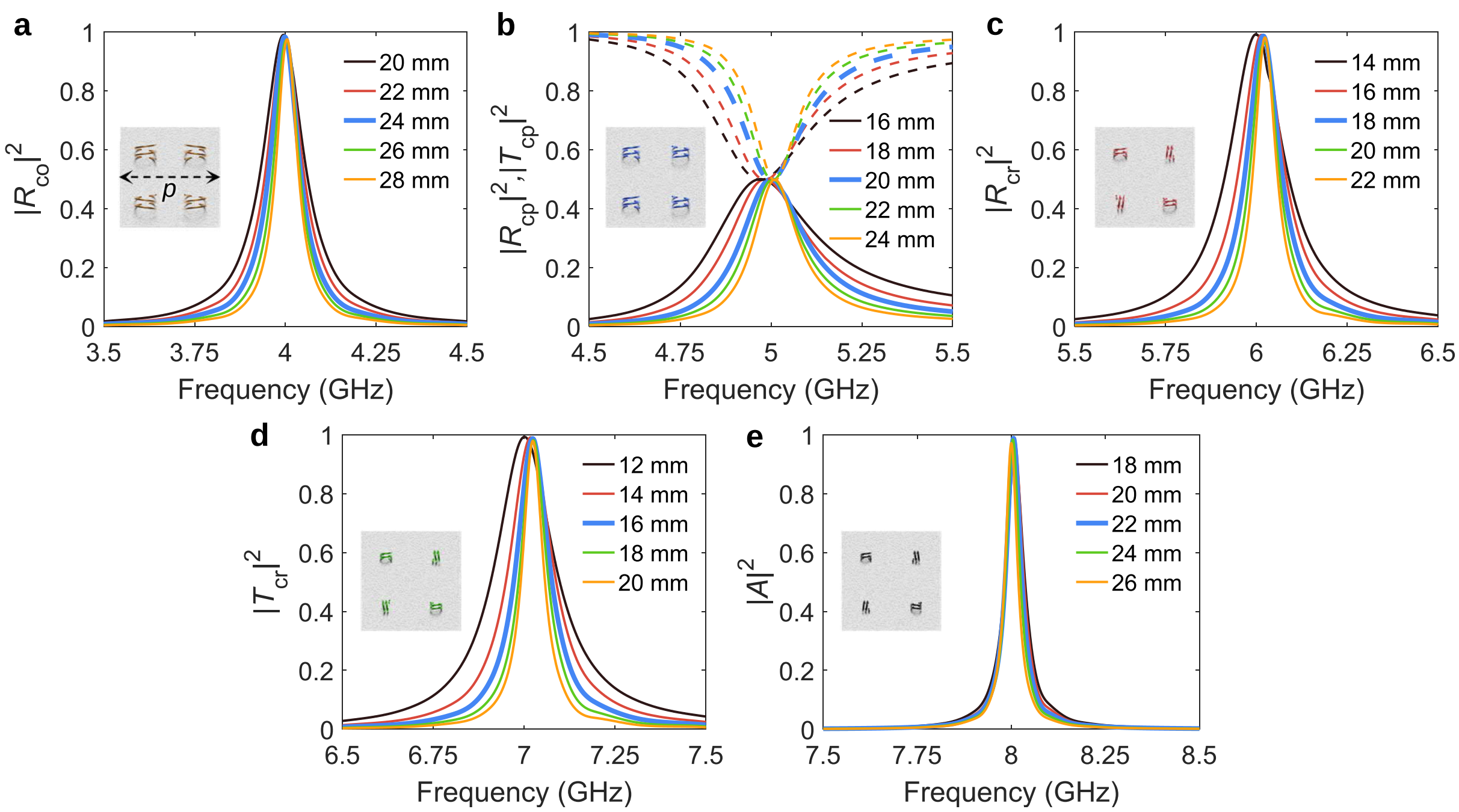

Figure S4: Dependence of the electromagnetic response of individual functional metasurfaces on the unit-cell period p. (a) Co-polarized reflection coefficient $|R_{\mathrm{co}}|^2$ for the MMSS. (b) Circular-polarized reflection and transmission coefficients $|R_{\mathrm{cp}}|^2$ and $|T_{\mathrm{cp}}|^2$ for the CPSS. (c) Cross-polarized reflection coefficient $|R_{\mathrm{cr}}|^2$ for the RPR. (d) Cross-polarized transmission coefficient $|T_{\mathrm{cr}}|^2$ for the TPR configuration. (e) Absorption coefficient $|A|^2$ for the PA. Insets show the corresponding unit-cell layouts. The optimal performance for each functionality is highlighted with a bold line.

Variations of p primarily affect the spectral position and bandwidth of the resonance, while the peak efficiency of each functionality remains close to unity. This behavior confirms that the individual metasurface responses are robust against moderate changes in

lattice spacing, enabling flexible geometric tuning without compromising performance. The insets in each panel show the corresponding unit-cell configurations used in the simulations.

Fig. S5 illustrates the influence of the inter-element distance on the performance, as an example, of the MMSS and PA metasurfaces. Panel (a) shows the unit-cell geometry of MMSS with different spacings between the helical resonators, while panel (b) presents the corresponding co-polarized reflection spectra. As the inter-element distance is varied, the resonance frequency mainly shifts at the shortest distance due to strong coupling of helices, whereas the peak reflection amplitude remains close to unity, indicating that the MMSS functionality is preserved.

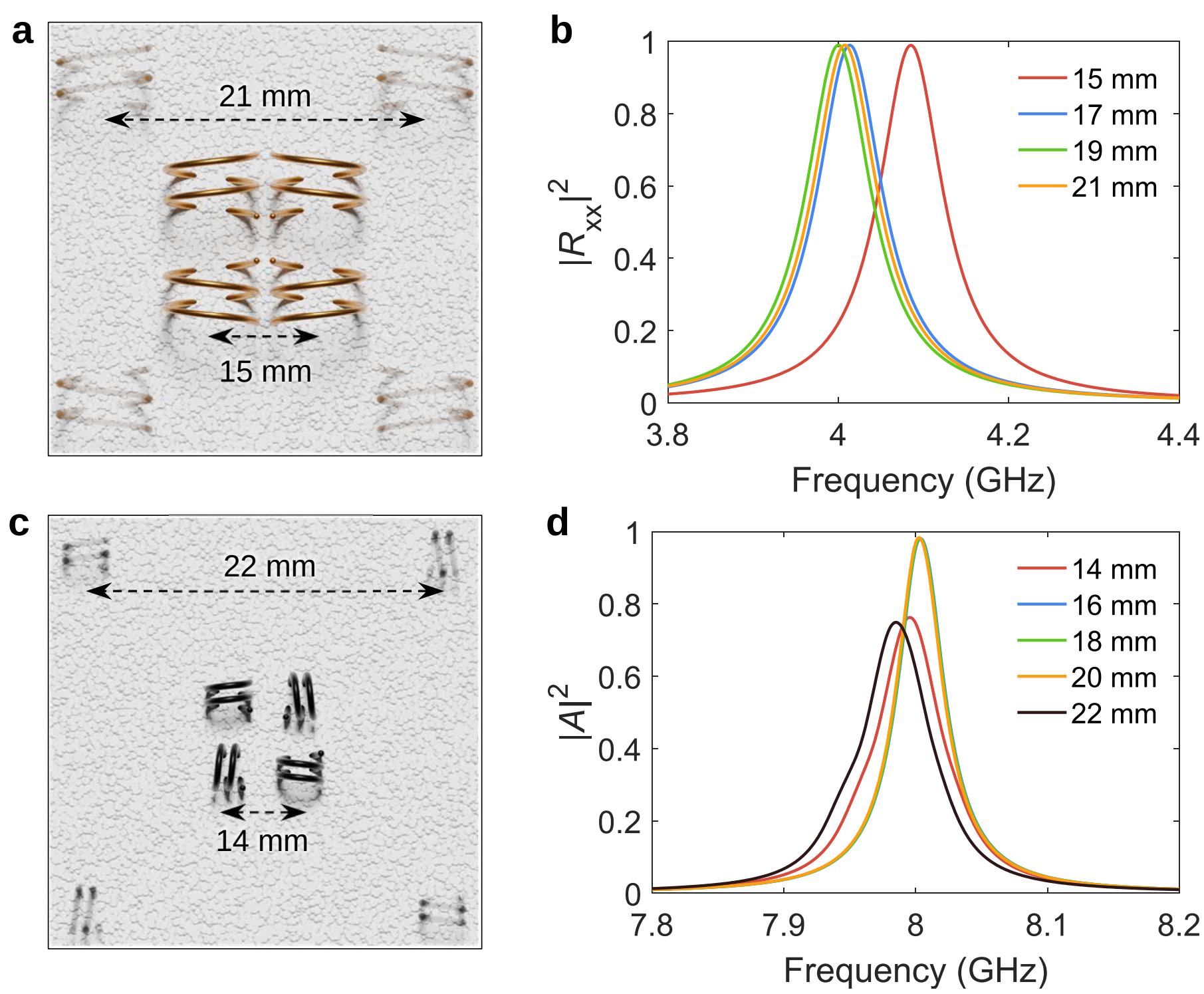

Figure S5: Effect of inter-element distance on the electromagnetic response of (a,b) the MMSS and (c,d) the PA metasurfaces.

Fig. S5(c,d) shows the unit-cell layout and the analogous analysis for the perfect absorber. As shown in Fig. S5(d), the resonance amplitude decreases for both smaller and

larger inter-element distances within the unit cell, while near-unity absorption is preserved when the elements are arranged close to a symmetric centered configuration. These results demonstrate that both MMSS and PA metasurfaces are robust against moderate variations in geometric spacing, enabling flexible tuning of the operational frequency without compromising functionality.

Fig. S6 presents the amplitude and phase characteristics of the five individual metasurface functionalities. Fig. S6(a,b) correspond to the magnetic mirror (MMSS), showing the co-polarized reflection coefficients and the associated reflection phase. The helix-based MMSS exhibits an almost polarization-independent response, and the reflection phase at resonance is close to 0°, which is characteristic of a magnetic mirror. Fig. S6(c,e,g,i) demonstrates that the CPSS, RPR, TPR, and PA metasurfaces exhibit polarization-independent electromagnetic responses throughout their respective resonance bands. The results demonstrate that each metasurface operates independently at its designed frequency and maintains polarization-independent amplitude responses for both x- and y-polarized linear excitation.

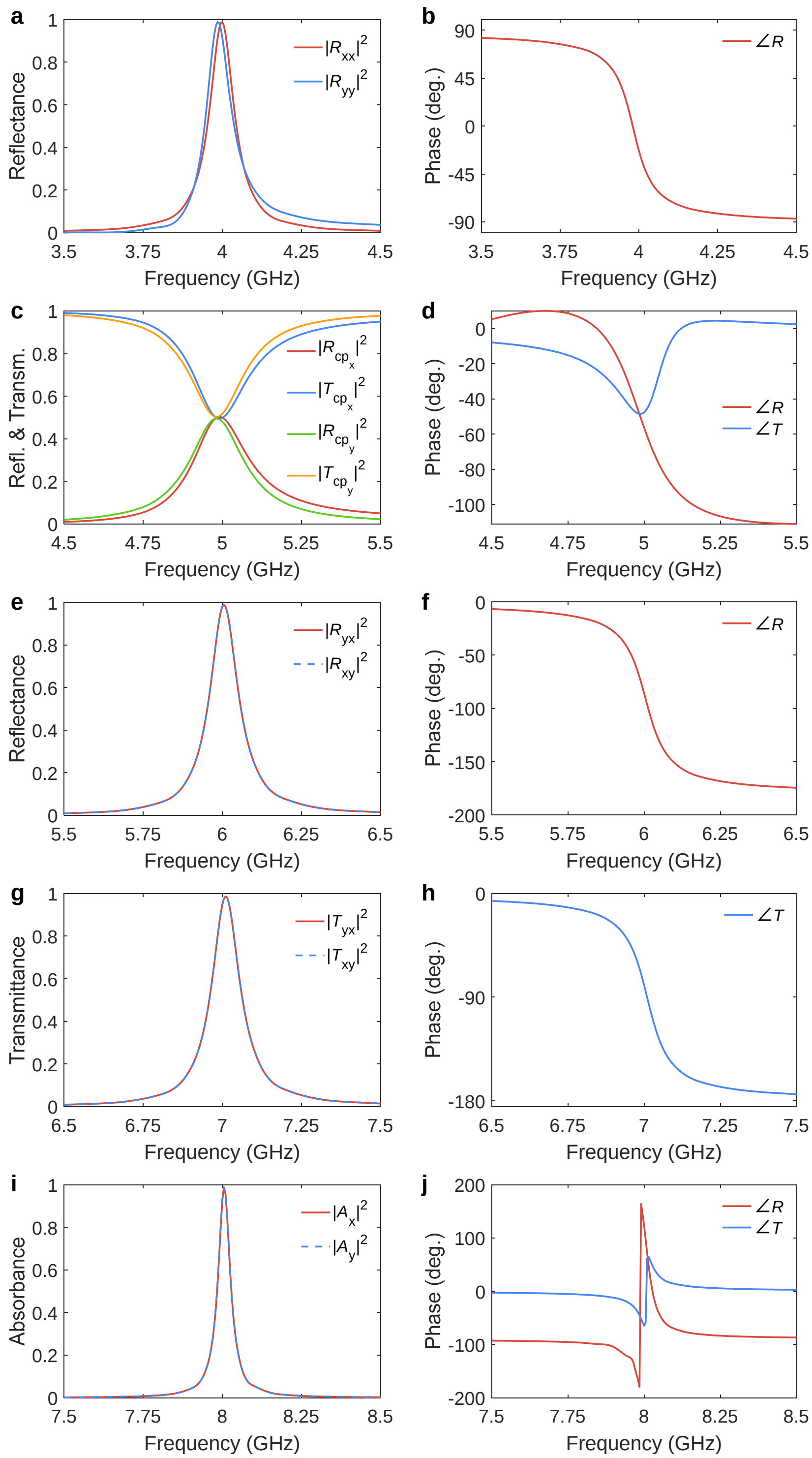

Figure S6: Amplitude and phase responses of the individual functional metasurfaces: (a,b) MMSS, (c,d) CPSS, (e,f) RPR, (g,h) TPR, and (i,j) PA, respectively.

Fig. S7 presents the polarization characteristics of the metasurfaces under linearly polarized excitation along the x- and y directions. The indices x (or xx, yy) denote co-polarization, while the indices xy or yx indicate cross-polarization of the incident wave. Fig. S7(a) shows the rotation angle and ellipticity of the reflected waves for the MMSS, demonstrating nearly linearly polarized reflection for both polarization states. Panel (b) illustrates the ellipticity of the reflected and transmitted waves for the CPSS, confirming circular polarization of reflected and transmitted waves at the resonance frequency.

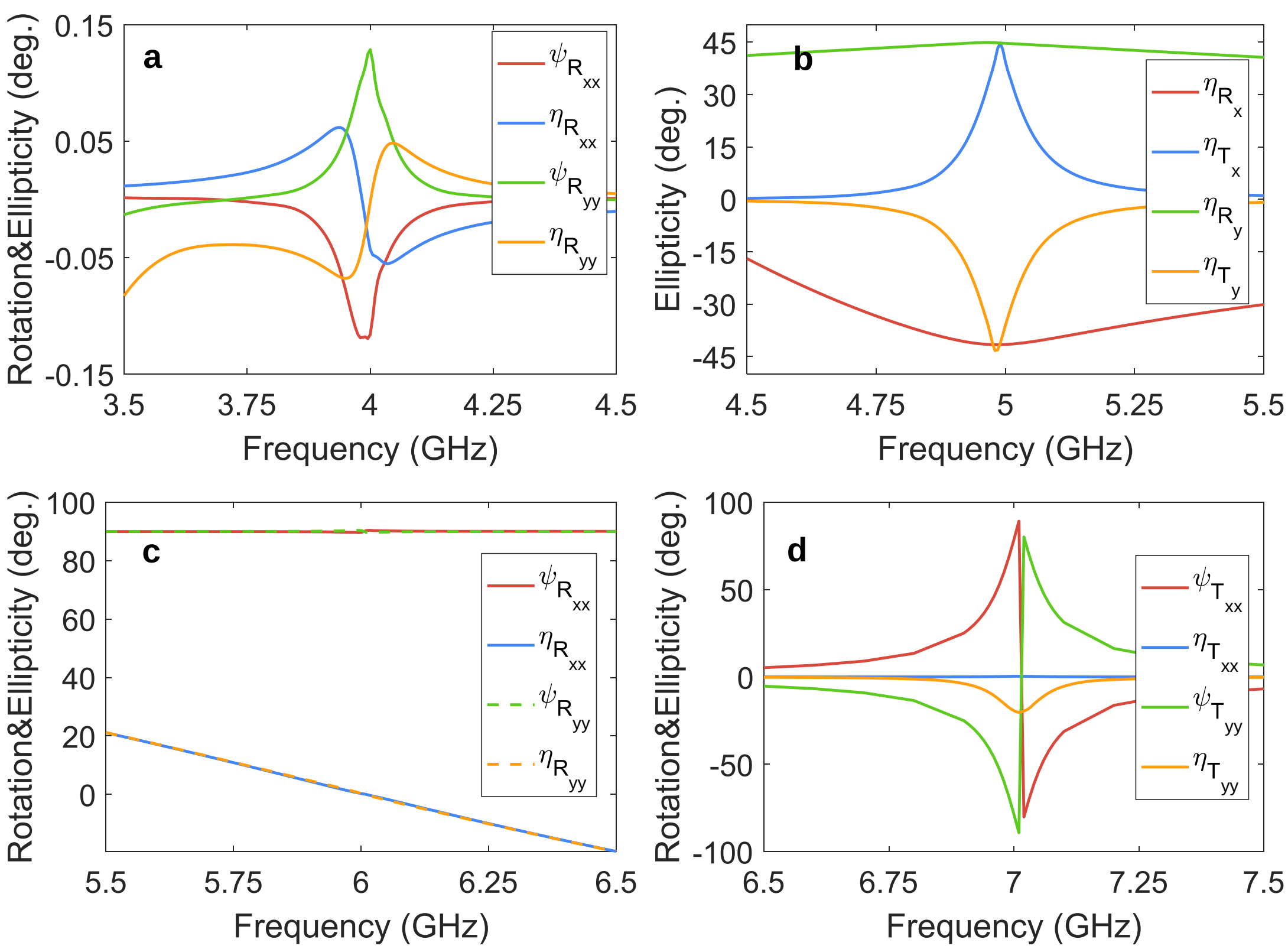

Figure S7: Polarization characteristics of the metasurfaces under x- and y-polarized linear excitation. (a) Rotation angle and ellipticity of the reflected waves for the MMSS. (b) Ellipticity of the reflected and transmitted waves for the CPSS. (c) Rotation angle and ellipticity of the reflected waves for the RPR. (d) Rotation angle and ellipticity of the transmitted waves for the TPR.

Fig. S7(c) displays the rotation angle and ellipticity of the reflected waves for the RPR, where polarization rotation by 90° is observed for x- and y-polarized excitation. A similar

polarization conversion is observed in transmission for the TPR, as shown in Fig. S7(d). Overall, these results confirm that the metasurfaces realize the intended polarization conversions at their resonance frequencies while maintaining consistent performance for both orthogonal linear polarizations of the incident wave.

4 Polarization-independence of the single-layer unit-cell metasurface

Fig. S8 summarizes the polarization-independent electromagnetic responses of individual single-layer metasurface unit cells under linearly polarized excitation along the x and y directions. Panel (a) shows the co-polarized reflection coefficients of the MMSS, where a slight shift in the resonance frequency is observed, while the reflection amplitude remains nearly identical for both polarizations, indicating polarization-independent behavior. Panel (b) presents the circularly polarized reflection and transmission coefficients of the CPSS, where equal splitting between reflection and transmission is observed for both excitation directions. Panels (c) and (d) display the cross-polarized reflection and transmission responses of the RPR and TPR, respectively, confirming identical conversion efficiencies. Finally, panel (e) shows the absorption spectra of the PA, where the absorption amplitude slightly decreases while the resonance frequency remains unchanged.

Overall, these results confirm that all individual unit cells of the single-layer metasurfaces maintain polarization-independent amplitude responses at their respective resonance frequencies, ensuring consistent electromagnetic functionality for orthogonal linear polarizations of the incident wave.

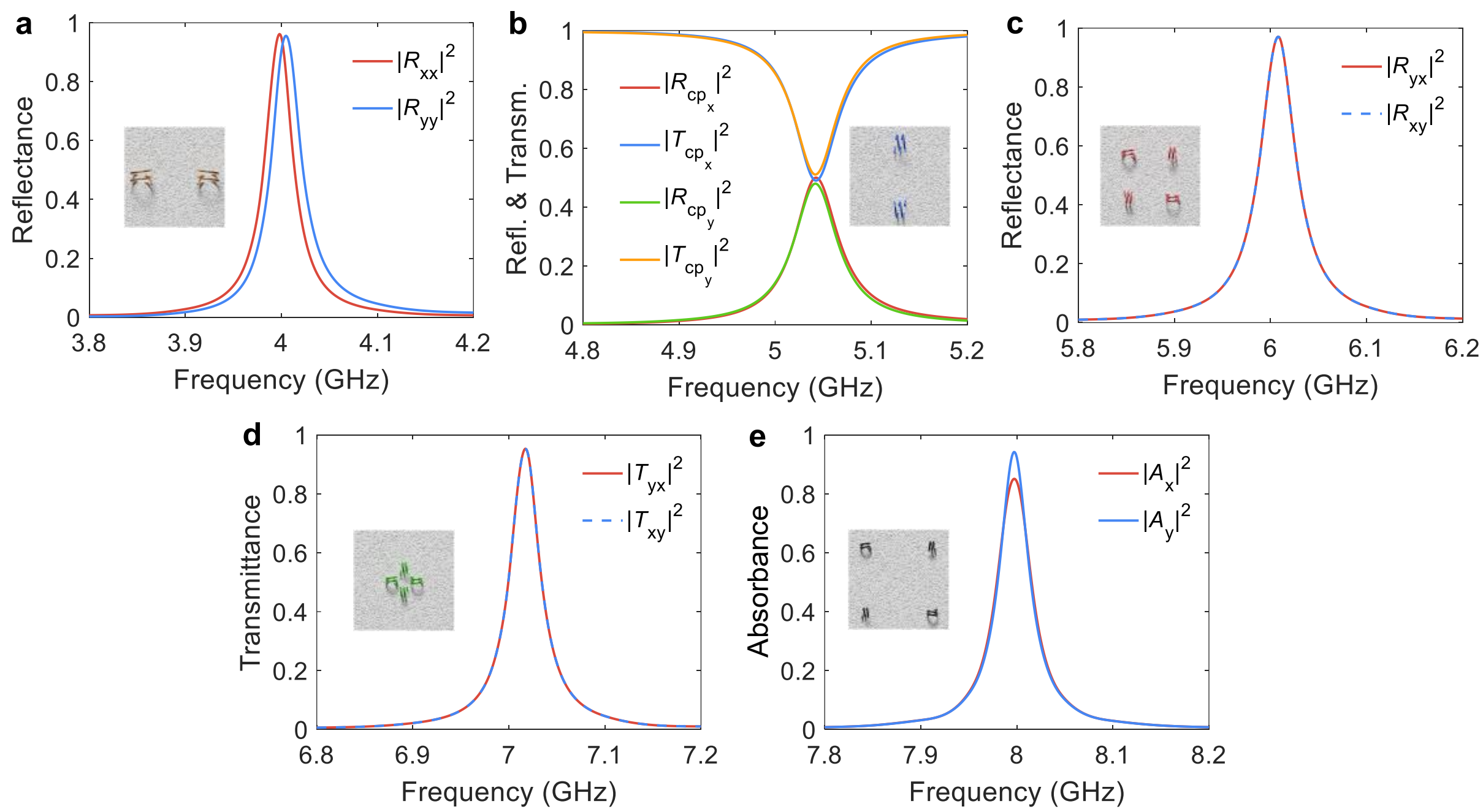

Figure S8: Polarization-independent electromagnetic responses of the single-layer metasurface unit cell under x- and y-polarized linear excitation. (a) Co-polarized reflection coefficients of the MMSS. (b) Circularly polarized reflection and transmission coefficients of the CPSS. (c) Cross-polarized reflection coefficients of the RPR. (d) Cross-polarized transmission coefficients of the TPR. (e) Absorption coefficients of the PA.

Fig. S9 compares the distributions of the electric field for the cascaded (multi-layer) and single-layer metasurface configurations at the operating frequencies of 4, 5, 6, 7, and 8 GHz. Fig. S9(a) shows the field distributions for the cascaded metasurface, where individual functional layers are spatially separated along the propagation direction. At each frequency, the electric field is predominantly localized around the corresponding functional layer, confirming that each layer independently performs its electromagnetic operation, while the remaining layers remain largely transparent.

Fig. S9(b) presents the corresponding electric field distributions for the single-layer metasurface. Despite the collapse of the cascaded structure into a single planar layer, the field localization remains strongly frequency selective. At each operating frequency, only the meta-atoms associated with the targeted functionality are efficiently excited, while

the other elements exhibit minimal interaction with the incident wave. The reflected and transmitted field components closely resemble those observed in the cascade metasurface.

Overall, the field distributions demonstrate that the single-layer metasurface successfully reproduces the functionality of the cascaded architecture, providing clear physical evidence that the cascade-to-single-layer transformation preserves the underlying electromagnetic mechanisms responsible for multifunctional operation.

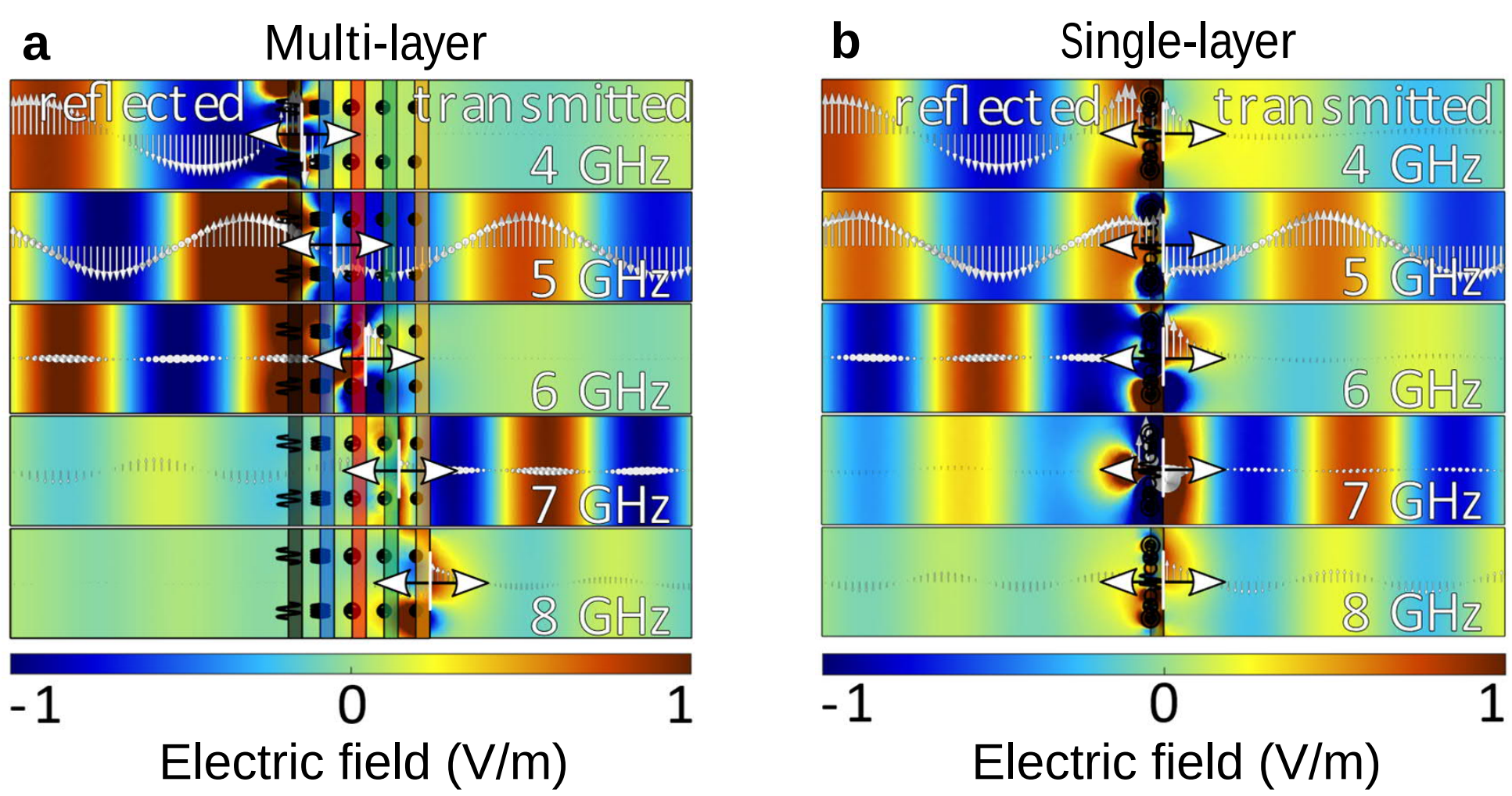

Figure S9: Electric field distributions illustrating the electromagnetic response of the (a) cascaded (multi-layer) and (b) single-layer helix-based metasurfaces at their respective operating frequencies. The field maps correspond to 4, 5, 6, 7, and 8 GHz, highlighting the reflected and transmitted wave components associated with each functional layer.

5 Robustness of single-layer multifunctional multispectral metasurface

Fig. S10 demonstrates that the single-layer metasurface retains its targeted electromagnetic functionalities even when they are realized at resonance frequencies different from those discussed in the main text. The helical meta-atoms are arranged at the same spatial positions within the unit cell as in Fig. 4(a) of the main text, while their geometric parameters are adjusted to shift the operating frequencies. In this configuration, the CPSS operates at 4 GHz, the MMSS at 5 GHz, the PA at 6 GHz, the RPR at 7 GHz, and the

TPR at 8 GHz.

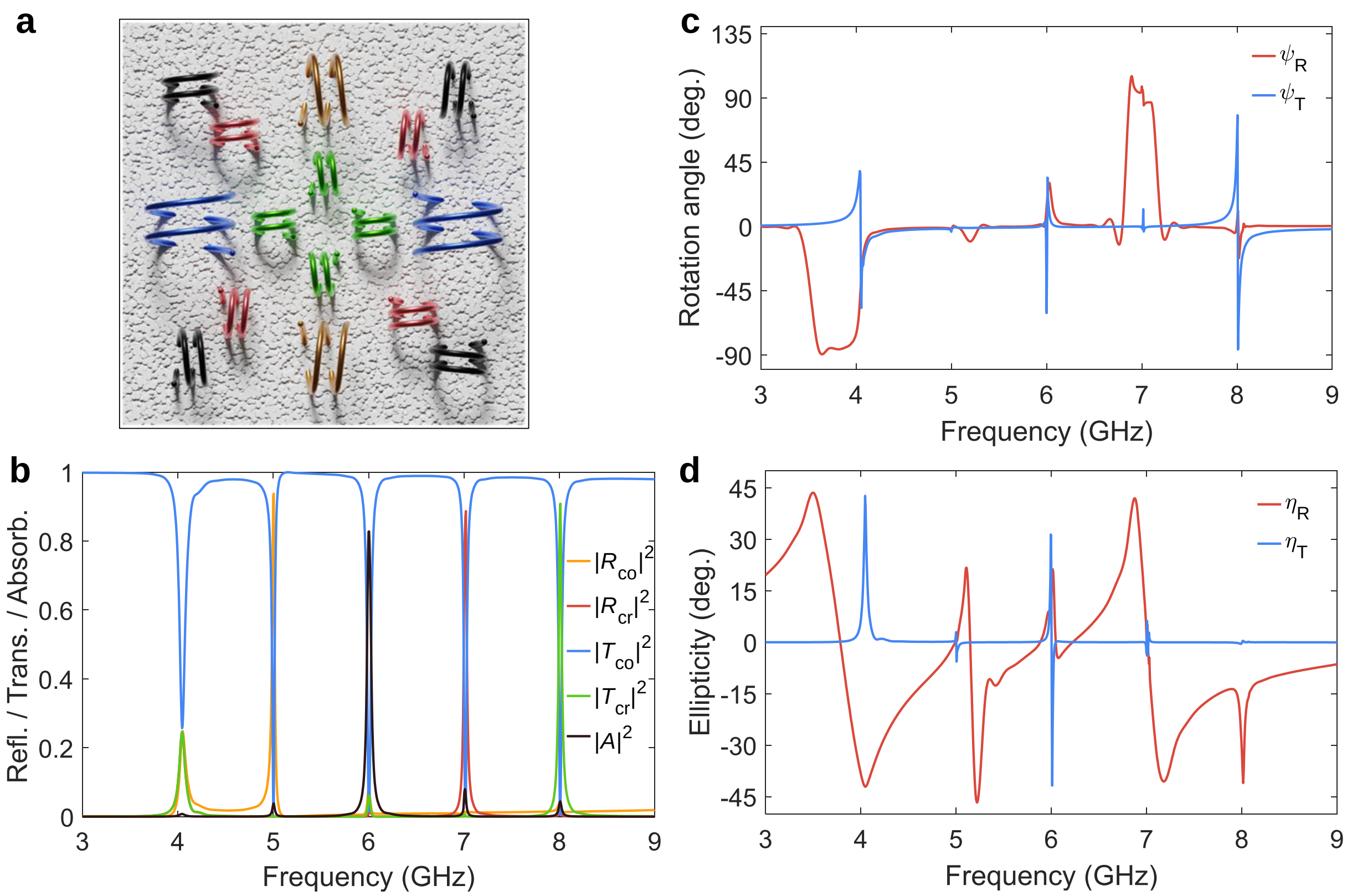

Figure S10: Electromagnetic response of the single-layer metasurface with corresponding functionalities at different resonant frequencies. (a) Unit cell of the metasurface. (b) Co- and cross-polarized reflection, transmission, and absorption spectra. (c) Polarization rotation angle and (d) ellipticity of the reflected and transmitted waves. Helices are placed at the same position within the unit cell as in the main text in Fig. 4(a).

Fig. S10(b) shows well-defined resonant features corresponding to the intended reflection, transmission, and absorption functionalities, indicating that the electromagnetic response remains robust. The associated polarization rotation and ellipticity responses shown in panels (c) and (d) further confirm that the desired polarization transformations are preserved.

These results indicate that the proposed single-layer metasurface architecture is flexible and scalable, allowing the operational frequencies of the individual functionalities to be modified without altering the spatial arrangement of the helical resonators within the unit

cell.

Fig. S11 presents the electromagnetic response of the single-layer metasurface with an alternative arrangement of helical resonators within the unit cell. The inter-element spacing of the helices is identical to that used in the main-text configuration shown in Fig. 4(a) and in Fig. S10(a).

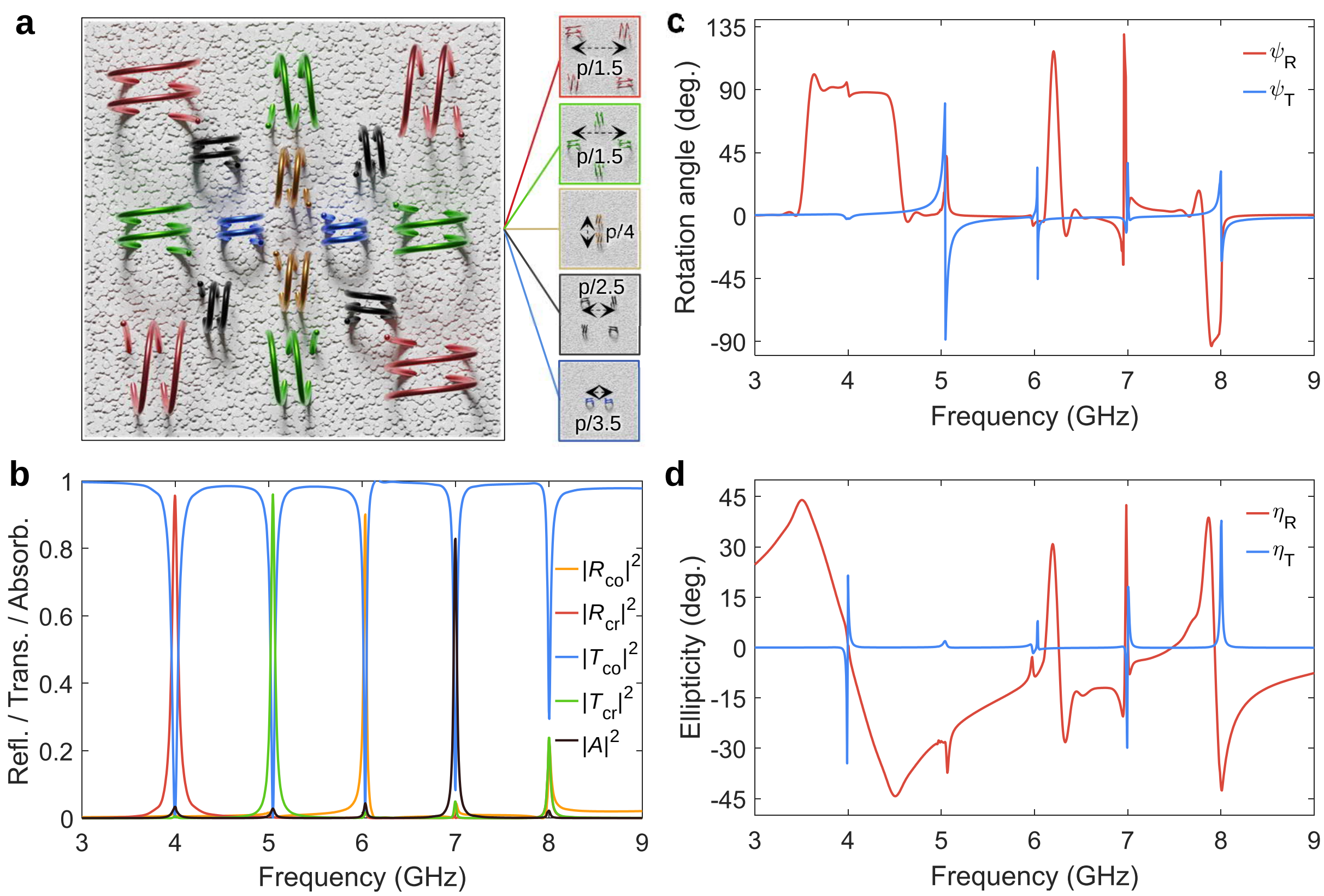

Figure S11: Electromagnetic response of the single-layer metasurface with an alternative arrangement of helical resonators within the unit cell. (a) Unit-cell showing modified spatial positions of the helices. (b) Co- and cross-polarized reflection, transmission, and absorption spectra corresponding to different metasurface functionalities operating at distinct frequencies. (c) Polarization rotation angle and (d) ellipticity of the reflected and transmitted waves. The inter-element distance of helices is the same as in the main text in Fig. 4(a) and Fig. S10(a).

Fig. S11(b) displays the normalized co- and cross-polarized reflection, transmission, and absorption spectra. Despite the altered spatial arrangement, the metasurface clearly exhibits well-defined resonant responses associated with the intended functionalities, which

are realized at frequencies different from those discussed in the main text. This confirms that the operational frequencies can be reassigned through geometric optimization without requiring a strict arrangement of helical resonators.

Panels (c) and (d) present the polarization rotation angle and ellipticity of the reflected and transmitted waves, respectively. The results demonstrate that the desired polarization transformations are preserved across the corresponding resonance frequencies, indicating robust multifunctional performance.

Overall, these results confirm that the proposed single-layer metasurface architecture is highly flexible, allowing both the spatial arrangement of helices and the operating frequencies of the individual functionalities to be modified while maintaining efficient electromagnetic response.

References

[S1] Viktar S Asadchy, Igar A Faniayeu, Younes Ra'di, and Sergei A Tretyakov. Determining polarizability tensors for an arbitrary small electromagnetic scatterer. *Photonics and Nanostructures-Fundamentals and Applications*, 12(4):298–304, 2014.

[S2] Younes Ra'Di, Viktar S Asadchy, and Sergei A Tretyakov. Total absorption of electromagnetic waves in ultimately thin layers. *IEEE Transactions on Antennas and Propagation*, 61(9):4606–4614, 2013.

[S3] John David Jackson. Classical electrodynamics, 1998.

[S4] Rasoul Alaee, Carsten Rockstuhl, and Ivan Fernandez-Corbaton. An electromagnetic multipole expansion beyond the long-wavelength approximation. *Optics Communications*, 407:17–21, 2018.

[S5] Ihar Faniayeu, Viktar Asadchy, and Ivan Fanyaev. Polarization control with helical metasurfaces. *Crystals*, 10(9):726, 2020.
